# P-1521. Prophylactic Immunization with Nirsevimab Does Not Impair the Development of Natural Antibody Responses to the Pre-fusion Conformation of the Respiratory Syncytial Virus Fusion Protein

**DOI:** 10.1093/ofid/ofaf695.1705

**Published:** 2026-01-11

**Authors:** Ann Marie Stanley, Vancheswaran Gopalakrishnan, Carolina Caceres, Kevin M Tuffy, Beth Kelly, Mark T Esser, Tonya L Villafana, Anastasia A Aksyuk, Deidre Wilkins

**Affiliations:** Translational Medicine, Vaccines & Immune Therapies, BioPharmaceuticals R&D, AstraZeneca, Gaithersburg, MD, USA, Gaithersburg, MD; AstraZeneca, Gaithersburg, MD; AstraZeneca, Gaithersburg, MD; AstraZeneca, Gaithersburg, MD; AstraZeneca, Gaithersburg, MD; AstraZeneca, Gaithersburg, MD; AstraZeneca, Gaithersburg, MD; Translational Medicine, Vaccines & Immune Therapies, BioPharmaceuticals R&D, AstraZeneca, Gaithersburg, MD, USA, Gaithersburg, MD; Translational Medicine, Vaccines & Immune Therapies, BioPharmaceuticals R&D, AstraZeneca, Gaithersburg, MD

## Abstract

**Background:**

Nirsevimab is an extended half-life (M252Y/S254T/T256E [YTE]-modified) monoclonal antibody that binds to the prefusion (pre-F) conformation of the respiratory syncytial virus (RSV) fusion (F) protein and is licensed for the prevention of RSV lower respiratory tract disease in neonates, infants and medically vulnerable children. Previous analyses have shown that nirsevimab immunization does not impair the development of antibody (Ab) responses to the G attachment, nucleocapsid or postfusion (post-F) conformation of the RSV F protein. Pre-F-specific Abs determine the magnitude of RSV neutralizing activity. However, the impact of nirsevimab immunization on pre-F responses has been difficult to assess due to shared epitopes. Here we describe a method of depleting YTE-modified Abs from serum, and its application in assessing natural pre-F binding and neutralizing Ab (nAb) responses following nirsevimab immunization in participants from the placebo-controlled Phase 3 MELODY study (NCT03979313).

**Figure:**
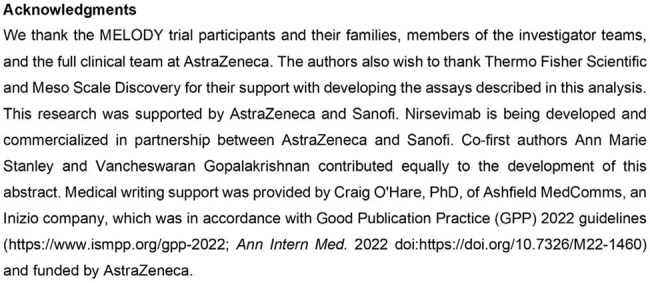

**Figure 1:**
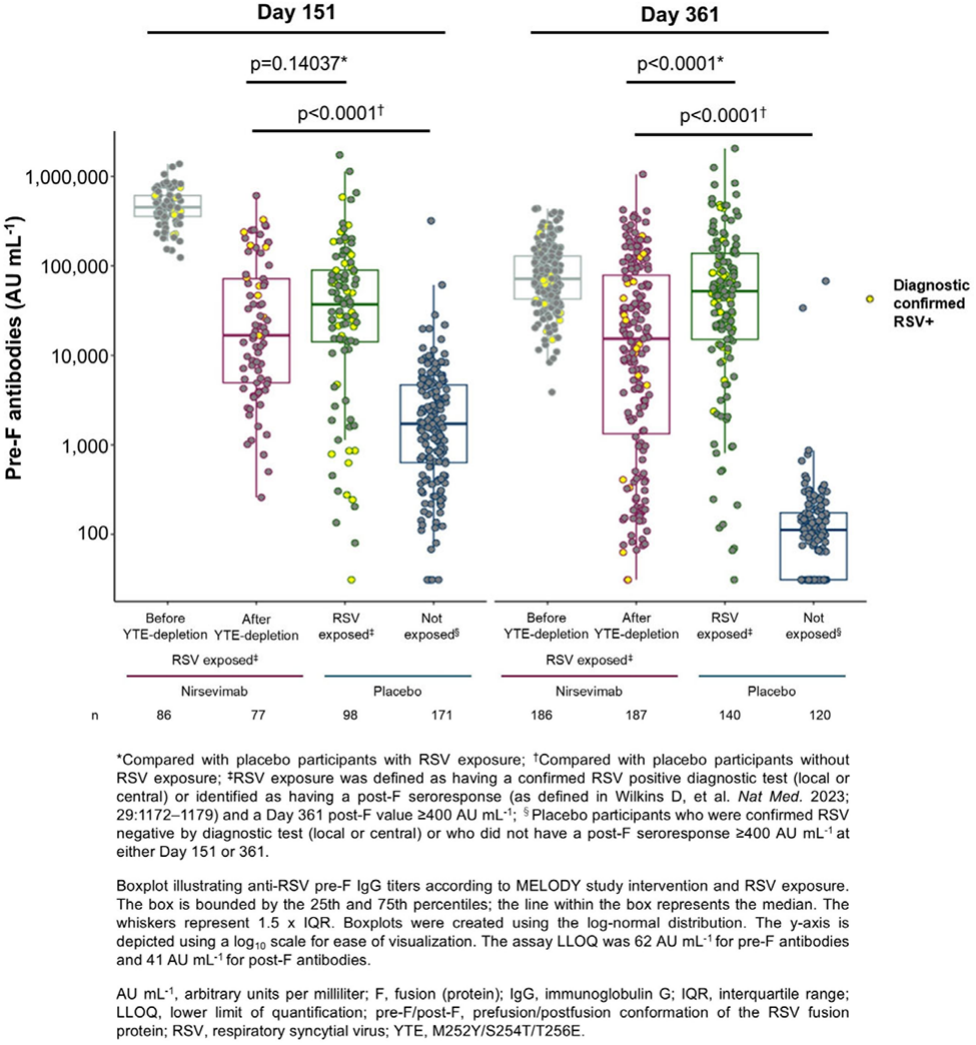

**Methods:**

Participants provided serum samples during site visits on Day 151 and 361. Nirsevimab was selectively depleted from serum using streptavidin beads coupled to an anti-YTE capture Ab. Pre-F Ab and nAb levels were respectively measured by multiplex serology and fluorescent focus-based microneutralization assay before and after YTE-depletion. Participant RSV exposure was determined by a diagnostic test or post-F seroresponse.

**Figure 2:**
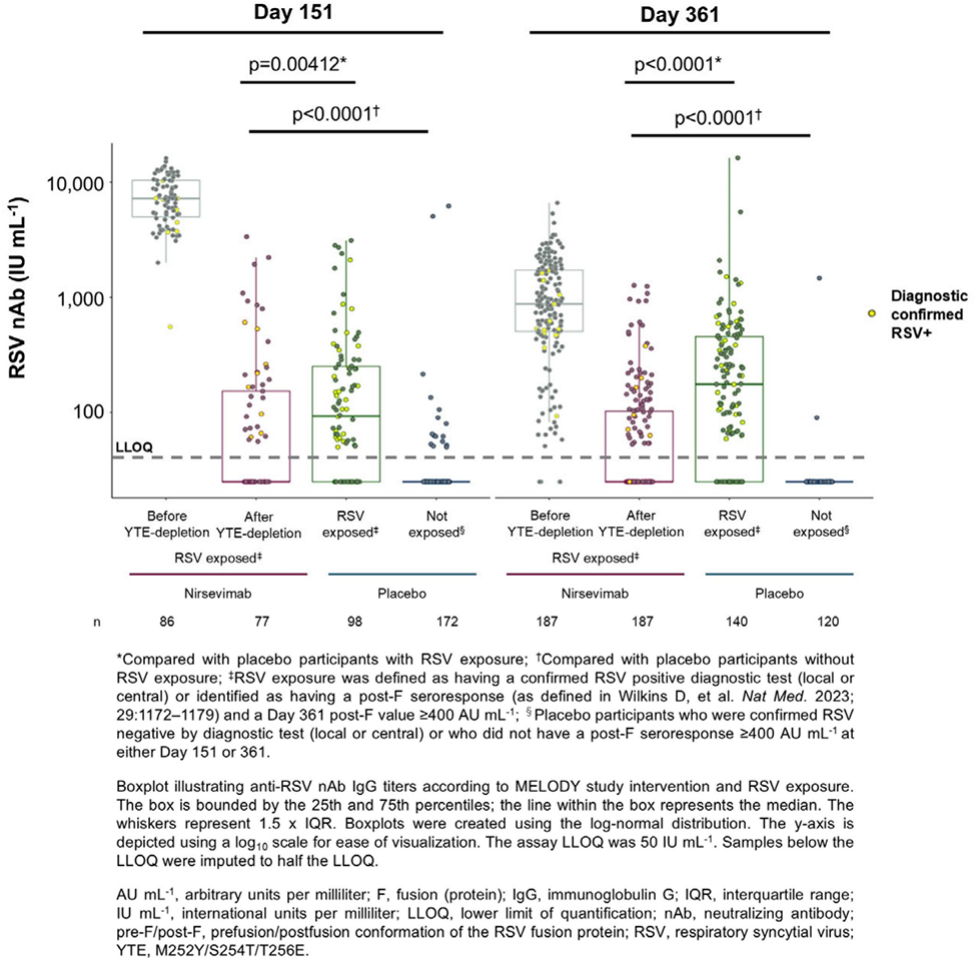

**Results:**

Pre-F Ab responses were induced in nirsevimab-immunized participants with diagnostically confirmed RSV exposure or post-F seroresponses (Figure 1). The range of pre-F Ab responses in nirsevimab-immunized participants overlapped with those from placebo participants with RSV exposure, albeit with lower median concentrations, and was higher than responses in placebo participants without exposure. Similar trends were observed for nAb responses above the assay’s lower limit of detection. The range of nAb responses in nirsevimab-immunized participants was similar, albeit lower, than observed in placebo participants with RSV exposure, and higher than in placebo participants without exposure (Figure 2).

**Conclusion:**

Nirsevimab immunization does not prevent an infant’s immune system from developing natural pre-F binding and nAb responses.

**Disclosures:**

Ann Marie Stanley, PhD, AstraZeneca: Employement|AstraZeneca: Stocks/Bonds (Public Company) Vancheswaran Gopalakrishnan, PhD, AstraZeneca: Employment|AstraZeneca: Stocks/Bonds (Public Company) Carolina Caceres, MS, AstraZeneca: Employment|AstraZeneca: Stocks/Bonds (Public Company) Kevin M. Tuffy, MS, AstraZeneca: Employment|AstraZeneca: Stocks/Bonds (Public Company) Beth Kelly, PhD, AstraZeneca: Employment|AstraZeneca: Stocks/Bonds (Public Company)|Sanofi: Employment|Sanofi: Stocks/Bonds (Private Company) Mark T. Esser, PhD, AstraZeneca: Employment|AstraZeneca: Stocks/Bonds (Public Company) Tonya L. Villafana, PhD, MPH, AstraZeneca: Employment|AstraZeneca: Stocks/Bonds (Public Company) Anastasia A. Aksyuk, PhD, AstraZeneca: Employment|AstraZeneca: Stocks/Bonds (Public Company)|MesoScale Diagnostics: Intellectual Property/Patents Deidre Wilkins, BSC, AstraZeneca: Employment|AstraZeneca: Stocks/Bonds (Public Company)

